# Nomogram based on blood lipoprotein for estimation of mortality in patients with hepatitis B virus-related acute-on-chronic liver failure

**DOI:** 10.1186/s12876-020-01324-w

**Published:** 2020-06-15

**Authors:** Cuicui Xiao, Jiao Gong, Shuguang Zhu, Zhiwei Zhang, Sujuan Xi, Yutian Chong, Yusheng Jie, Qi Zhang

**Affiliations:** 1 Cell-gene Therapy Translational Medicine Research Center, Key Laboratory of Liver Disease of Guangdong Province, Third Affiliated Hospital of Sun Yat-sen University, Guangzhou, People’s Republic of China; 2 Department of Laboratory Medicine, Third Affiliated Hospital of Sun Yat-sen University, Guangzhou, People’s Republic of China; 3 Department of Hepatic Surgery and Liver Transplantation Center, Third Affiliated Hospital of Sun Yat-sen University, Guangzhou, People’s Republic of China; 4 Department of Infectious Diseases, Key Laboratory of Liver Disease of Guangdong Province, Third Affiliated Hospital of Sun Yat-sen University, Guangzhou, People’s Republic of China

**Keywords:** Nomogram, Acute-on-chronic liver failure, Hepatitis B virus, Low-density lipoprotein

## Abstract

**Background:**

Acute-on-chronic liver failure (ACLF) is a clinic syndrome with substantial high short-term mortality. It is very important to stratify patients according to prognosis to decide management strategy. This study aimed to formulate and validate a nomogram model based on blood lipoprotein for prediction of 3-month mortality in patients with hepatitis B virus (HBV)-related ACLF.

**Methods:**

Data on 393 consecutive patients who were diagnosed as HBV-related ACLF at the Third Affiliated Hospital of Sun Yat-sen University between June 1, 2013, and February 1, 2015, were prospectively collected. Of these, 260 patients who were collected in an earlier period formed the training cohort for the development of nomogram, while 133 patients who were collected thereafter formed the validation cohort for confirming the performance of nomogram.

**Results:**

Multivariate analysis showed that low density lipoprotein cholesterol (LDL-C), age, prothrombin time, and creatinine were independently associated with 3-month mortality of patients with HBV-related ACLF. Kaplan–Meier survival analysis revealed that the high LDL-C (LDL-C ≥ 1.0 mmol/L, cut-off value) was significantly associated with elevated overall survival (*P* < 0.001). All independent factors for survival were selected into the nomogram. The calibration plot for the probability of survival showed good agreement between prediction by nomogram and actual observation.

**Conclusion:**

This study highlighted that reduction of serum LDL-C level was an independent risk factor for the survival in patients with HBV-related ACLF, and the nomogram based on serum LDL-C was an accurate and practical model for predicting the 3-month mortality in patients with this disease.

## Background

Acute-on-chronic liver failure (ACLF) is a syndrome featured by acute deterioration of chronic liver disease associated with hepatic and extrahepatic organ failures, and also associated with substantial high short-term mortality [1–3]. Alcohol and chronic viral hepatitis are the most common causes underlying liver diseases. Data from the CANONIC study indicated that overall 28-day mortality is 33%, and 3-month mortality is 51% in all cases of ACLF, and ACLF grade at diagnosis is significantly associated with short-term prognosis [4, 5]. Thus, it is very crucial to stratify patients according to prognosis, in order to choose the treatment strategy.

Unfortunately, until recently, model for end-stage liver disease (MELD) scores, and Child-Pugh-Turcotte scores, the conventional scoring systems to evaluate prognosis in patients with cirrhosis, were the only available tools to assess prognosis in patients with ACLF [6]. However, these scores have limited accuracy to predict prognosis in ACLF, as they do not reflect the systemic inflammation, which is the characteristic pathophysiological feature of this syndrome [7, 8]. It is reported that sepsis with multiple organ failure, also characterized by systemic inflammation, is frequently associated with a substantial reduction of circulating lipoprotein levels [9, 10]. However, there are very limited evidences of the alteration of circulating lipoprotein levels in ACLF.

Due to the lack of a specific and practical predictive tool, development of a predictive model that incorporates factors associated with ACLF based on diagnostic clinic pathologic data becomes intensively desirable. Of all the available models, a nomogram can provide an evidence based, individualized, highly accurate risk estimation [11, 12]. In addition, nomograms are easy to use and can facilitate therapy-related decision making. In this study, we established a nomogram based on blood lipoprotein for predicting 3-month mortality in hepatitis B virus (HBV)-related ACLF, which is the primary cause of ACLF in China.

## Methods

### Patients

Between June 1, 2013, and February 1, 2015, data on consecutive patients who had HBV-related ACLF were prospectively collected at the Third Affiliated Hospital of Sun Yat-sen University. This study was approved by the Institutional Ethics Committee of the Third Affiliated Hospital of Sun Yat-sen University. Informed consent was obtained from all enrolled patients for their data to be used for this study. Patients did not receive any financial compensation.

Consensus recommendations of the Asian Pacific Association for the Study of the Liver (APASL) 2014 for ACLF were used as the diagnostic criteria in this study [13]. The inclusion criteria were (1) a history of chronic hepatitis B (positive HBsAg > 6 months); (2) a history of liver cirrhosis, diagnosed by clinical (stigmata), biochemical (decreased serum albumin and increased serum globulin levels), and imaging findings (ultrasound, computed tomography or magnetic resonance imaging); (3) evidence of liver failure, existing at least one of the following situation: serum total bilirubin (TBIL) level > 5 times of the normal level, i.e., TBIL ≥85 μmol/L; prothrombin activity < 40% of the normal level; hepatorenal syndrome; progressive reduction in liver size; ascites; or hepatic encephalopathy. Patients who received steroids within 6 months before admission, had a history of infection with hepatitis C virus, hepatitis D virus, hepatitis E virus, human immunodeficiency virus (all these viruses were detected by enzyme linked immunosorbent assay kit), or autoimmune liver disease were excluded. 260 eligible patients enrolled between June 1, 2013, and September 1, 2014, were included into the training cohort for the development of a nomogram, and 133 eligible patients enrolled between September 1, 2014, and February 1, 2015, were entered into the validation cohort.

### Laboratory examination

Fasting blood samples were collected from all patients within 24 h after admission. The blood biochemical examinations included complete blood count, renal function (creatinine), liver function [albumin, alanine aminotransferase (ALT) and TBIL], and lipid [total cholesterol (TC), triglyceride (TG), high-density lipoprotein cholesterol (HDL-C), low-density lipoprotein cholesterol (LDL-C)], and coagulation [prothrombin activity (PTA) and international normalized ratio (INR)]. All biochemical parameters were detected by standard automated laboratory methods and using commercially available kits according to the manufacturers protocols. In details, blood cell count was analyzed on the Sysmex XN-9000 hematology analyzer; ALT, AST, albumin, TC, and TG were measured using the detection kit (Sichuan Maccura Biotechnology) and analyzed by HITACHI 7600–020; LDL-C, HDL-C, and creatinine were measured using a detection kit (Sekisui Medical) and analyzed by HITACHI 7600–020; the PTA and INR were evaluated by the STAGO full automatic analyzer.

### MELD score

MELD score was utilized to evaluate the disease severity at admission using a mathematical formula based on three lab parameters: TBIL, the international normalized ratio (INR) for prothrombin time, and creatinine levels. The MELD score was computed using a web calculator (http://www.mayoclinic.org/gi-rst/mayomodel7.html).

### Statistical analysis

Continuous variables are expressed as mean ± standard error of the mean (SEM), and compared using the Student’s *t* test or Mann-Whitney *U* test. Categorical variables were compared using the χ^2^ test or Fisher exact test. Survival curves were analyzed using the Kaplan-Meier method and compared using the log-rank test. Multivariate Cox regression analysis was used to evaluate the independent risk factors of 3-month mortality.

The significance of each variable in the training cohort was evaluated by univariate Cox regression analysis for determining the independent risk factors of 3-month mortality. All variables associated with 3-month mortality at a significant level were candidates for stepwise multivariate Cox regression analysis. A nomogram was formulated based on the results of multivariate Cox regression analysis and by using the rms package of R, version 3.4.0 (http://www.r-project.org/). The predictive performance of the nomogram was measured by Harrell’s concordance index (C-index), calibrated with 1000 bootstrap samples, and assessed by comparing nomogram-predicted vs observed Kaplan–Meier estimated survival probability. In addition, comparisons between the nomogram and MELD score were performed by the C-index with the rcorrp.cens package in *Hmisc* in R.

In all analyses, *P* < 0.05 was considered to be statistical significance. All analyses were performed using SPSS, version 15.0 (SPSS Inc., Chicago) or R, version 3.4.0.

## Results

### Clinicopathologic characteristics of patients

During the study period, 403 consecutive patients were diagnosed as HBV-related ACLF. Of these, 393 patients who met the inclusion criteria were enrolled. 260 and 133 patients were divided into the training and validation cohorts, respectively. Besides, the median follow-up time was 63 ± 30 days and 71 ±  33 days in the training and validation cohorts, respectively. Survival rate of all patients at 3-month was 70.99%. The clinicopathologic characteristics of all the enrolled patients were listed in Table 1. The baseline of clinicopathologic data were similar between the training and validation cohorts.

**Table 1:** The baseline characteristics of patients with ACLF

	Development group (***n*** = 260)	Validation group(***n*** = 133)	***p***
Age (year)	45 ± 12	45 ± 13	0.571
Gender (male/female)	222/38	108/25	0.310
Red blood cell count (× 10^12^/L)	3.93 ± 0.91	3.88 ± 0.95	0.620
White blood cell count (×10^9^/L)	8.08 ± 3.56	9.43 ± 7.78	0.058
Hemoglobin (g/dL)	119.92 ± 23.35	118.58 ± 22.86	0.587
Albumin (g/L)	31.76 ± 4.54	31.42 ± 4.32	0.469
Alanine aminotransferase (U/L)	707.38 ± 877.45	649.70 ± 855.85	0.534
Prothrombin activity (%)	29.28 ± 7.57	27.87 ± 7.84	0.085
INR	2.75 ± 1.00	3.05 ± 1.27	0.019
Creatinine (μmol/L)	78.27 ± 44.77	85.84 ± 70.73	0.262
Total bilirubin (μmol/L)	378.86 ± 168.36	376.05 ± 158.08	0.871
TC (mmol/L)	2.44 ± 1.31	2.42 ± 1.05	0.849
TG (mmol/L)	0.86 ± 0.52	0.96 ± 0.51	0.056
HDLC (mmol/L)	0.19 ± 0.18	0.18 ± 0.17	0.546
LDLC (mmol/L)	1.10 ± 0.60	1.06 ± 0.65	0.568
MELD scores, median (IQR)	25.98 (23.13, 29.4)	26.46 (24.16, 31.19)	0.055
Ascites (yes/no)	167/93	95/38	0.175
mortality (yes/no)	77/183	37/96	0.726

*Abbreviations*: *INR* international normalized ratio, *TC* total cholesterol, *TG* triglyceride, *HDL-C* high-density lipoprotein cholesterol, *LDL-C* low-density lipoprotein cholesterol

### The optimal cut-off value of LDL-C for predicting survival in ACLF

All variables listed in Table 1 were used for univariate and multivariate Cox regression analysis. The results of univariate analysis of overall survival were reported in Table S1. In multivariate analysis, reported as hazard ratio and 95% confidence interval (CI), the result showed that LDL-C (0.731; 95% CI, 0.541–0.987), age (1.031; 95% CI, 1.013–1.050), PTA (0.945; 95% CI, 0.917–0.975), and creatinine (1.004; 95% CI, 1.000–1.008) were independently associated with 3-month mortality of patients with ACLF (Table 2). These results indicated that LDL-C was a protective factor for the survival in ACLF.

**Table 2:** Multivariate analysis of the training cohort

Variable^a^	HR	95% CI	***p***
LDL-C	0.731	0.541–0.987	0.041
Age	1.031	1.013–1.050	0.001
Prothrombin activity	0.945	0.917–0.975	< 0.001
Creatinine	1.004	1.000–1.008	0.037

*Abbreviations*: *HR* hazard ratio, *CI* confidence interval

^a^Variables in this analysis included gender, age, prothrombin time, LDL-C, TBIL, albumin, creatinine

**Fig. 1:**
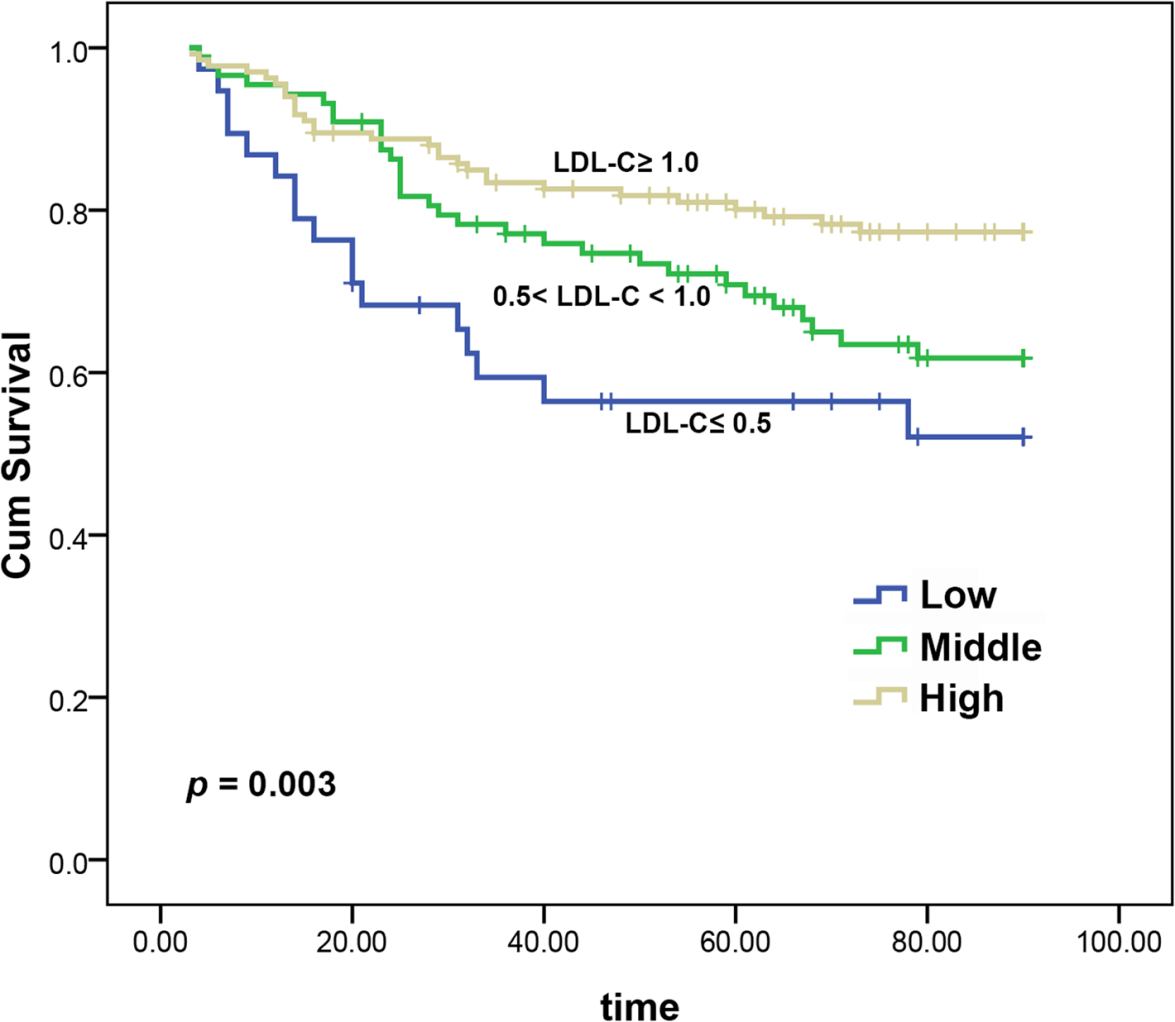
Kaplan-Meier survival curves of the training cohort categorized by the serum LDL-C level (mmol/L)

X-tile software was used to determine the optimal cut-off values of LDL-C for predicting overall survival, which was 0.5 mmol/L and 1.0 mmol/L. Patients were divided into low group (LDL-C ≤ 0.5 mmol/L), middle group (0.5 < LDL-C < 1.0 mmol/L), and high group (LDL-C ≥ 1.0 mmol/L) for further analysis. Kaplan–Meier survival analysis revealed that the high LDL-C level was significantly associated with elevated overall survival (*p* = 0.003) (Fig. 1).

### Development and validation of a survival-predicting nomogram

**Fig. 2:**
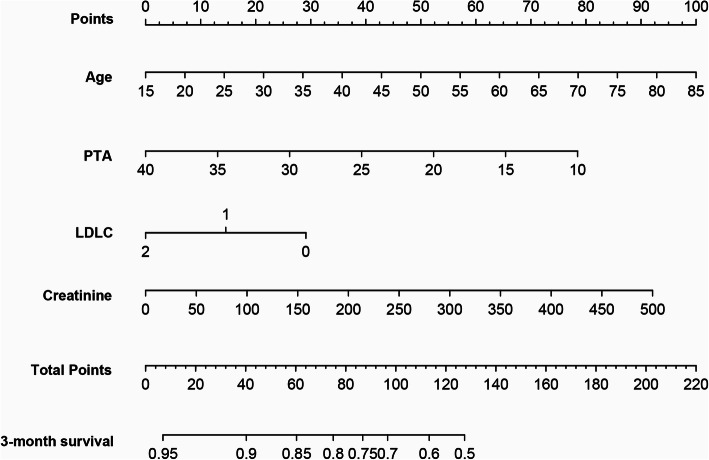
Nomogram to estimate the 3-month survival probability in HBV-related ACLF. To use this nomogram, find the position of each variable on the corresponding axis, then draw an upward line to the points axis for the value of points. Add the points from all of the variables, and draw a downward line from the total points axis to determine the probability of 3-month survival at the lower line of the nomogram

**Fig. 3:**
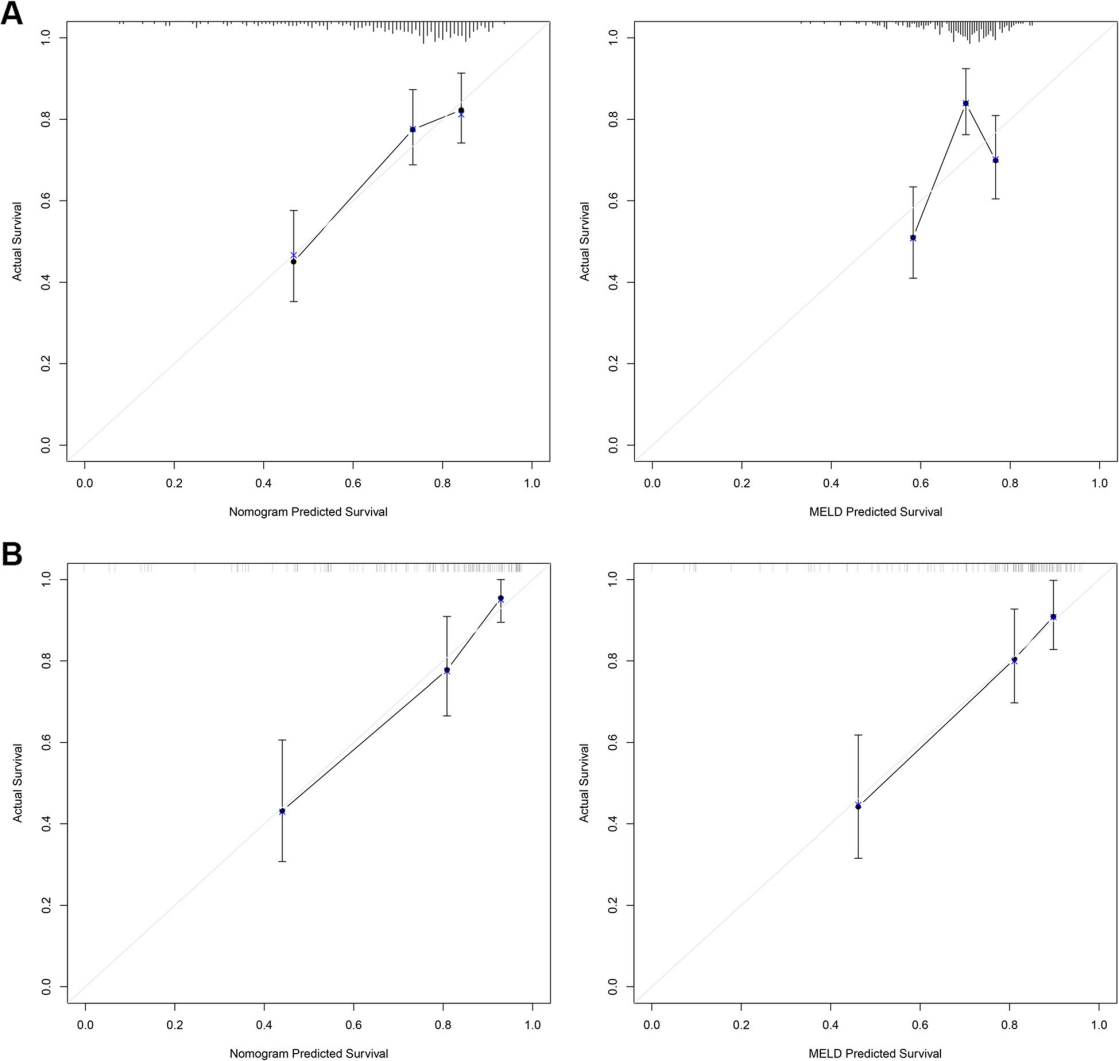
The predictive performance of the nomogram. (**a**) Validity of the predictive performance of the nomogram and MELD score in estimating the risk of mortality in the training cohort. (**b**) Validity of the predictive performance of the nomogram and MELD score estimating the risk of mortality in the validation cohort. Nomogram or MELD score predicted probability of overall survival is plotted on the x-axis, while actual overall survival is plotted on the y-axis

All independent factors were used to construct a 3-month mortality risk estimation nomogram (Fig. 2). The resulting model was internally validated using the bootstrap validation method. The nomogram demonstrated good accuracy in estimating the risk of 3-month mortality, with a Harrell’s C-index of 0.700 (95% CI, 0.640–0.760), which was markedly higher than the MELD score (0.591; 95% CI, 0.515–0.667; *p* < 0.001). In addition, the calibration plot graphically displayed a good agreement on the probability of 3-month mortality between the prediction by nomogram and actual observation (Fig. 3a). In the validation cohort, the nomogram showed a C-index of 0.794 (95%CI, 0.730–0.851) for the estimation of 3-month mortality, which is higher than MELD score (0.767; 95% CI, 0.702–0.841). There was also a good calibration curve for the mortality estimation in the validation cohort (Fig. 3b).

### Risk of mortality based on nomogram scores

**Fig. 4:**
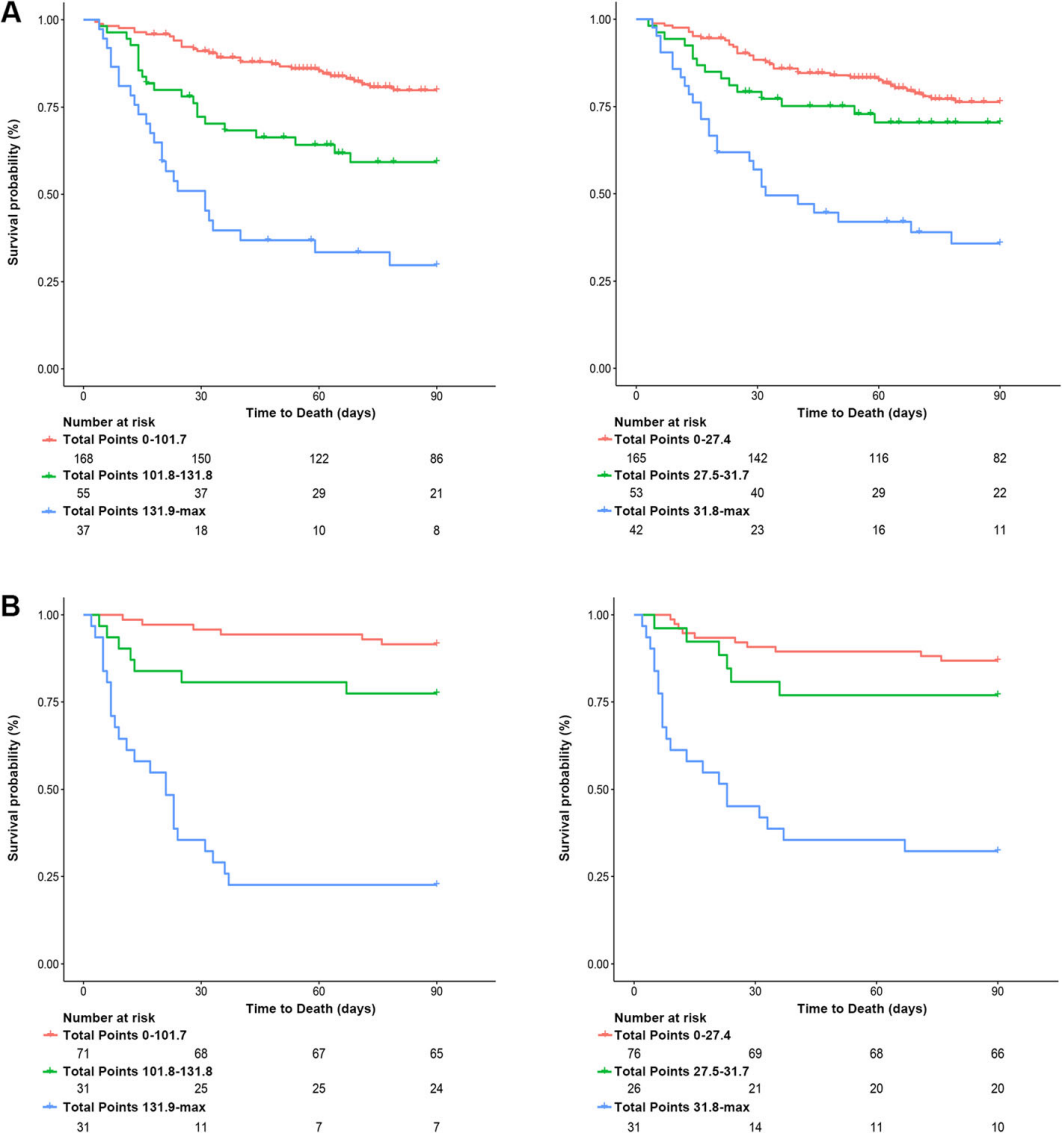
Kaplan-Meier survival curves of the two cohorts categorized by the nomogram (**a**) or MELD score (**b**)

For predicting the risk of 3-month mortality, the optimal cutoff value of total nomogram scores was determined to be 101.7 and 131.8. Accordingly, patients were divided into low risk group (0–101.7), middle risk group (101.7–131.8), and high risk group (≥131.8). Kaplan–Meier survival analysis revealed that each group represented a distinct prognosis in both the training cohort and validation cohort (Fig. 4a). Meanwhile, patients were also divided into three groups based on MELD scores using X-tile software, which is low risk group (≤27.4), middle risk group (27.5–31.7), and high risk group (≥31.8) (Fig. 4b). However, as shown in Fig. 4a and b, nomogram score was better than MELD score in analysis of the prognosis of patients in both cohorts.

## Discussion

Although the exact mechanism of ACLF still remains elusive, it is widely accepted that systemic inflammation is a hallmark of ACLF. Plasma levels of C reactive protein (CRP), white cell count, and pro-inflammatory molecules such as IL-1β and IL-6 are proved to be higher in the serum of patients with ACLF than in those without [8]. Therefore, it is reasonable that inflammatory biomarkers can predict the outcome of this dismal disease. In the previous study, we demonstrated that increased levels of neutrophil to lymphocyte ratio, an indicator of systemic inflammation, positively correlated with the poor prognosis of patients with ACLF [14]. In this study, our data showed that LDL-C levels were negatively correlated with the mortality of patients with ACLF. Importantly, a nomogram model based on LDL-C could accurately predicted the prognosis of patients with ACLF.

Reduction of blood lipoprotein during inflammatory diseases has been already observed in clinic for a long time. In 1911, Chauffard et al. reported diminished serum cholesterol levels in the febrile phase of tuberculosis, especially in patients with “very bad general condition” [9]. In addition, recent studies reported reduction of LDL-C level was an independent risk factor of in-hospital mortality in patient with acute heart failure [15–17]. In acute liver failure and acute on chronic liver failure, blood lipids, especially HDL, were significantly lower in patients who died or required a liver transplant [18, 19]. In this study, we reported that lower serum LDL-C level predicted lower rates of survival in patients with ACLF. One of the theories for explaining the benefit of high level of LDL-C for patient with heart failure is the “endotoxin lipoprotein hypothesis” [20]. It states that lipoproteins are needed to bind endotoxin and decrease the systemic release of pro-inflammatory cytokines, which results from the bacterial translocation from bowel. This theory may also account for the mechanism in ACLF, in which the situation of mesenteric venous and bowel wall edema is more serious than that in heart failure. However, the decreased levels of LDL-C might simply be an effect of advanced function failure of liver, which is the main source of LDL-C. Alternatively, studies have indicated that circulating cytokines may directly act to decrease lipoprotein levels by inhibiting hepatic lipoprotein production and promoting LDL receptor activity [9].

Owing to the substantial short-term mortality, it is very important to stratify patients according to prognosis, in order to decide allocation in the intensive care unit, or determine emergency for liver transplantation. Unfortunately, there are still lack of specific and practical predictive tools for this dismal disease. Nomograms are proved to be easy to use and can facilitate management-related decision making for many type of diseases. In this study, we formulated and validated a nomogram model consisting of age, PTA, LDL-C, and creatinine, which could be used to prognosticate 3-month mortality of patients with HBV-related ACLF. This nomogram score tool have several favorable characteristics. First, it is very simple to facilitate the clinical use. Second, parameters used in this tool is easy to obtain in the standard hospitalization. Third, the cut-off levels for the definition of disease grade have prognostic significance. Finally, it clearly improves the predictive ability of the currently available tools such as MELD score.

## Conclusions

Our study showed that reduction of serum LDL-C level was an independent risk factor for the survival in patients with HBV-related ACLF, and the nomogram based on serum LDL-C was an accurate and practical model for predicting the 3-month mortality in patients with this disease. However, a larger cohort might still need to validate the conclusion in this study.

## Supplementary information

**Additional file 1: Table S1.** Univariateanalysis of the primary cohort.

## Data Availability

All data generated or analyzed during this study are included in this article.

## Abbreviations

ACLF: Acute-on-chronic liver failure
HDL-C: High-density lipoprotein cholesterol
HBV: Hepatitis B virus
HR: Hazard ratio
LDL-C: Low-density lipoprotein cholesterol
MELD: End-stage liver disease
TBIL: Total bilirubin
TC: Total cholesterol
TG: Triglyceride
