# Supplementary material for: Nomogram based on blood lipoprotein for estimation of mortality in patients with hepatitis B virus-related acute-on-chronic liver failure
Source: BMC Gastroenterol. 2020 Jun 15;20:188. doi: 10.1186/s12876-020-01324-w (PMC7294630; doi:10.1186/s12876-020-01324-w)
Supplement: Supplementary file 1 — Additional file 1: Table S1. Univariateanalysis of the primary cohort. [file 12876_2020_1324_MOESM1_ESM.doc]

| **Table S1**. Univariateanalysis of the primary cohort | | |
| --- | --- | --- |
|  | **HR(95%CI)** | ***p*** |
| Age (year) | 1.036 (1.017-1.054) | 0.000 |
| Gender (male/female) | 0.529 (0.308-0.906) | 0.021 |
| White blood count (x10^9^/L) | 1.019 (0.958-1.084) | 0.541 |
| Hemoglobin (g/dL) | 0.994 (0.984-1.003) | 0.193 |
| Blood platelet (x10^9^/L) | 0.999 (0.995-1.003) | 0.509 |
| Albumin (g/L) | 0.935 (0.890-0.982) | 0.007 |
| Alanine aminotransferase (U/L) | 1.000 (1.000-1.000) | 0.832 |
| HDLC(mmol/L) | 1.036 (0.297-3.606) | 0.956 |
| LDLC(mmol/L) | 0.609 (0.454-0.816) | 0.001 |
| TC(mmol/L) | 0.937 (0.760 -1.157) | 0.547 |
| TG(mmol/L) | 0.710 (0.432-1.167) | 0.176 |
| Prothrombin activity (%) | 0.940 (0.912-0.968) | 0.000 |
| International normalized ratio | 1.295 (1.070-1.568) | 0.008 |
| Creatinine(μmol/L) | 1.004 (1.001-1.007) | 0.011 |
| Total bilirubin (μmol/L) | 1.001 (1.000-1.002) | 0.146 |

Abbreviations:TC, total cholesterol; TG, triglyceride; HDL-C, high-density lipoprotein; LDL-C, low-density lipoprotein.
